# Micro-Ureteroscopy as a Treatment of Renal Pelvis Lithiasis in Young Children

**DOI:** 10.3389/fped.2021.593743

**Published:** 2021-05-10

**Authors:** Alberto Parente, Ruben Ortiz, Beatriz Fernández-Bautista, Laura Burgos, José Maria Angulo

**Affiliations:** ^1^Reina Sofia University Hospital, Cordoba, Spain; ^2^Gregorio Marañón University Hospital, Madrid, Spain

**Keywords:** microperc, ureteroscopy, lithiasis in children, lithiasis, children

## Abstract

**Aim:** Miniaturization of endoscopic instruments has allowed to improve the efficacy of kidney stone treatment in young children. Aim of the study is to evaluate the usefulness of microureteroscopy with 4.85F sheath in the treatment of renal stones in children.

**M&M:** We present 4 cases of microureteroscopy in 3 younger than 3 years patients with renal pelvic stones. Lithiasis was unilateral in 2 female patients and bilateral in 1 male patient. Microureteroscopy was performed using a MicroPerc set 4.85F sheath, without placing a safety guide or dilating the meatus in 3 procedures. The lithotripter system used was Ho:YAG Laser with 200-μm fiber in all cases.

**Results:** Mean operating time was 100 ± 16 min. There were no intraoperative complications. In all procedures, complete pulverization of the lithiasis was achieved, except in the coraliform lithiasis in which 5 mm residual lithiasis remained in the lower calyx. All patients were discharged 24 h after the intervention.

**Conclusion:** Microureteroscopy can be considered a new alternative for treatment of selected cases of renal pelvic stones in infants and children.

## Introduction

Urolithiasis is an increasing common problem in pediatric age, so, we frequently diagnose more and younger children in our routine clinical assistance. Urolithiasis treatment may involve at least one of the following approaches: retrograde intrarenal surgery (RIRS) using flexible ureteroscopy, extracorporeal shock wave lithotripsy (ESWL) and percutaneous nephrolithotomy (PCNL) (1, 2), similar to adult urology. For this reason, we must adjust instruments available to younger children.

The main objective of kidney stone treatment is to achieve a total disappearance of the lithiasis with minimally invasive procedures. Treatment can include some combined procedures. however, the small size of the patient becomes a therapeutic challenge. ESWL needs one or two sessions to obtain satisfactory results, requiring general anesthesia and hospital admission in children. RIRS presents a technical difficulty due to the use of the smaller ureteroscopes necessary in pediatric patients, being also an expensive procedure. The use of PCNL can be invasive and associates some morbidity in children (3).

In 2011, Desai et al. (4) described a new set of instruments, initially designed to allow adequate renal access in PCNL under optical control. The objective was to achieve calyceal puncture and sheath placement in a single step. Therefore, we were able to reduce damage to renal parenchyma by percutaneous access. The procedure, which can use 2 types of small needles (8 Fr or 4.85 F), has been called “microperc” because of its small size. Subsequently, other authors presented their experience adding a three-way adapter that allows introducing a laser guide or fiber through it, in addition to the optic fiber and saline solution to distend the renal pelvis (5).

The main endourology advances is the miniaturization of the endoscopic instruments without losing its effectiveness. The use of smaller ureteroscopes can prevent ureteral meatus dilation and potential complications. Therefore, it reduces the ureteral damage, as well as its bleeding or rupture and the necessity of postoperative double J stent placement. Some urologists publications describe the treatment of pelvic distal ureteral lithiasis in women by retrograde microureteroscopy, with 4.85 Fr sheath used by micropercutaneous approach. Subsequently, some cases of ureteral lithiasis in young children have been reported using the 4.8 Fr sheath as a ureteroscope (6).

The aim of the study is to evaluate the usefulness of microureteroscopy in the treatment of kidney stones in small children.

## Materials and Methods

We present 3 younger 3 years old patients treated at our institution between 2017 and 2019. All lithiasis diagnoses were detected in the pyelonephritis evaluation. The lithiasis was unilateral in two female patient and bilateral in 1 male patient. A total of 4 microureteroscopies were performed, since in one of the patients a treatment of both kidneys was carried out in two surgical times.

Informed and written consent was obtained from the patients or their legal guardian in this study.

**Figure 1:**
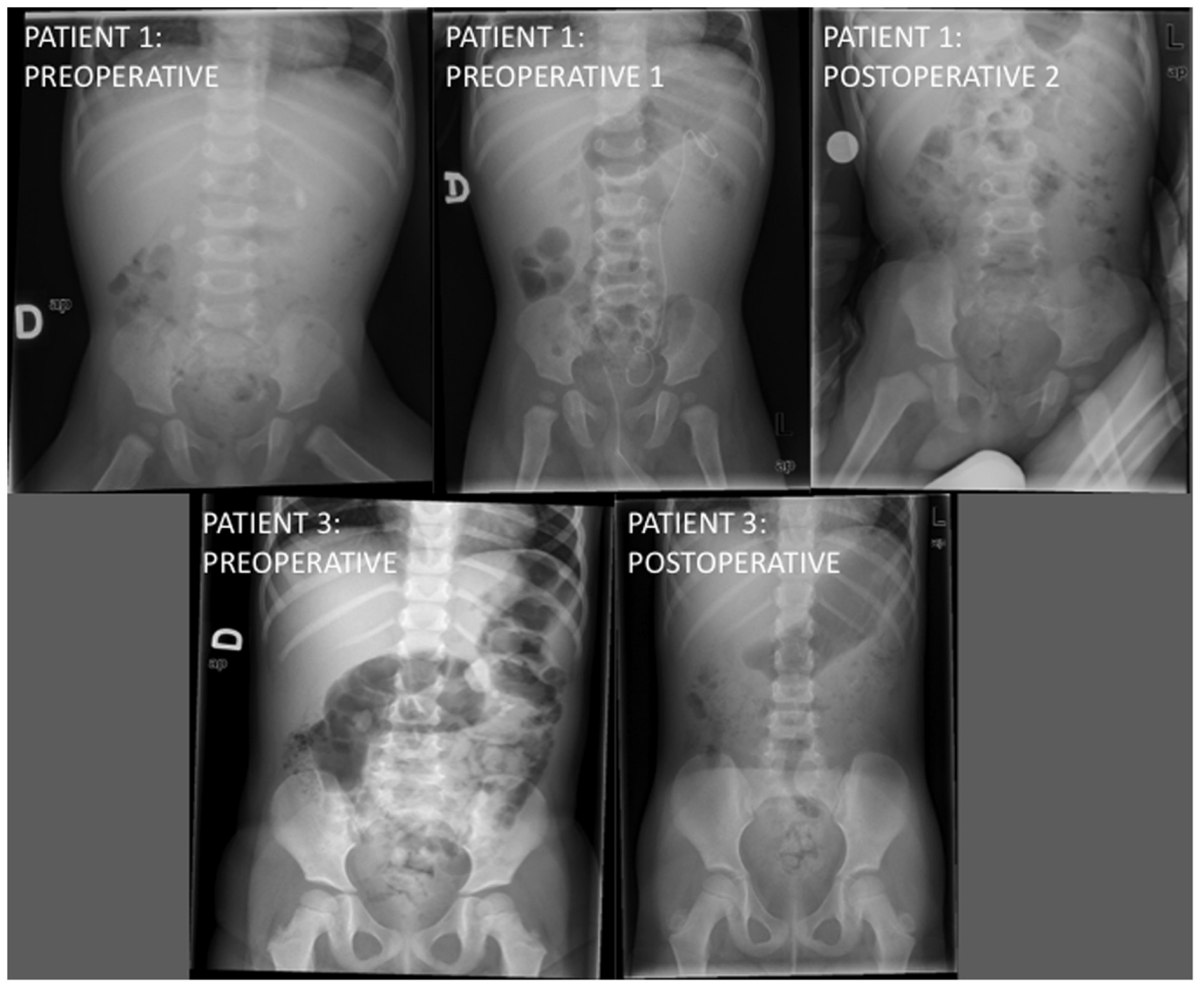
Radiological images of the first and third patients before and after microureteroscopy.

The male patient was 12 months and the female patients were 2 and 3 years. There were no previous diagnoses. In all cases, we found hydronephrosis (II to IV) of the affected kidney and lithiasis located in the renal pelvis. One of the girls had another two small lithiasis in the middle and inferior calyx. The size of the lithiasis were 10 to 23 mm. Radiological test showed a calcium density in two patients (Figure 1).

**Figure 2:**
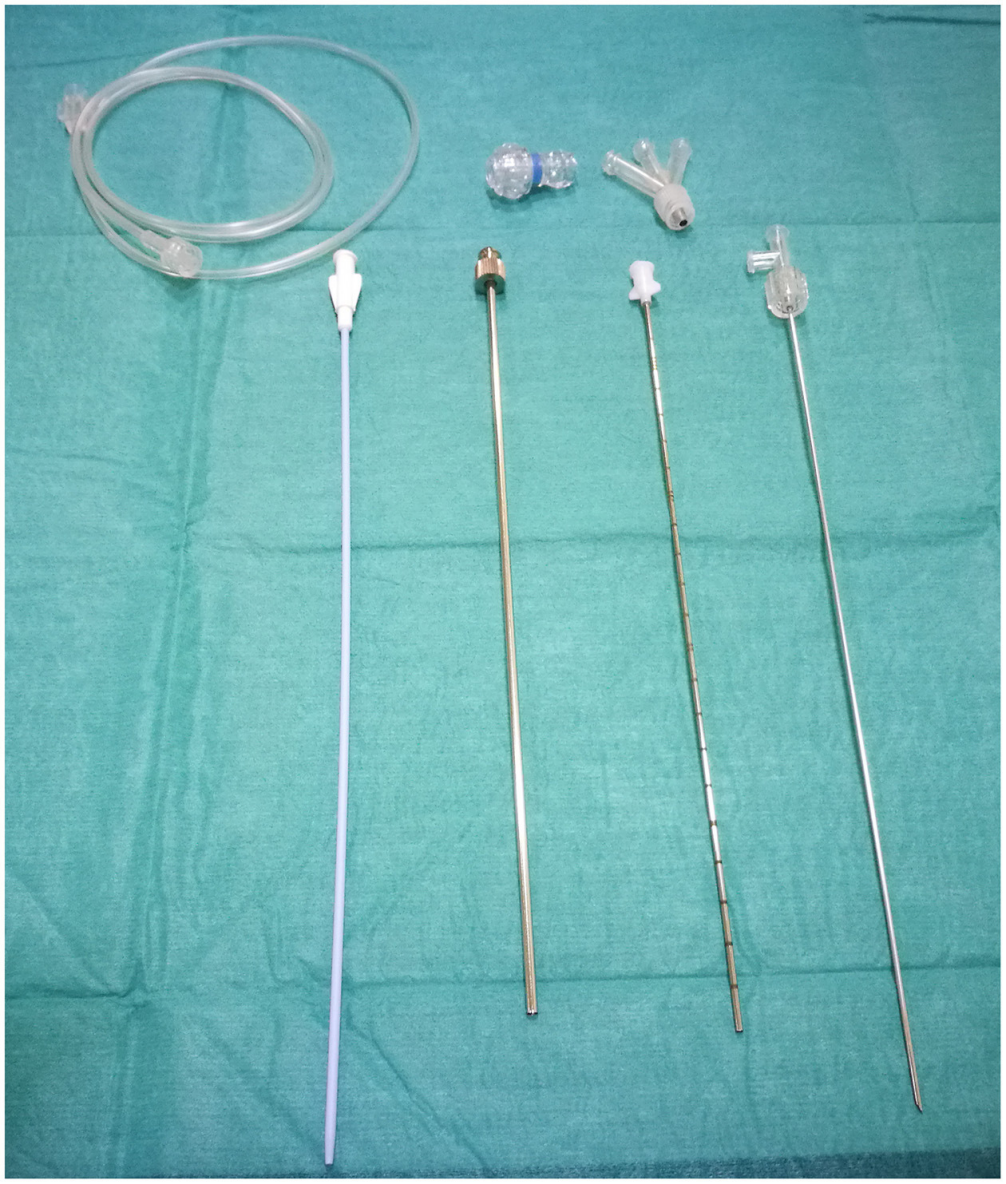
MicroPerc system set.

Microureteroscopy was performed using a MicroPerc set 4.85 F sheath (Figure 2). After approaching the ureteral meatus, microureteroscopy was accomplished without placing a safety guide or dilating the meatus in three procedures. The ureteroscopy began with a simple and virtually atraumatic passage through the meatus and intramural ureter. In one patient, high-pressure balloon dilatation was performed because pyeloureteral junction obstruction (PUJO) was observed during microureteroscopy. In this patient, microureteroscopy was performed with safety guide because of passage to renal pelvis was difficult by PUJO. So, it was performed using a hydrophilic guide to avoid damaging the pyeloureteral junction.

**Figure 3:**
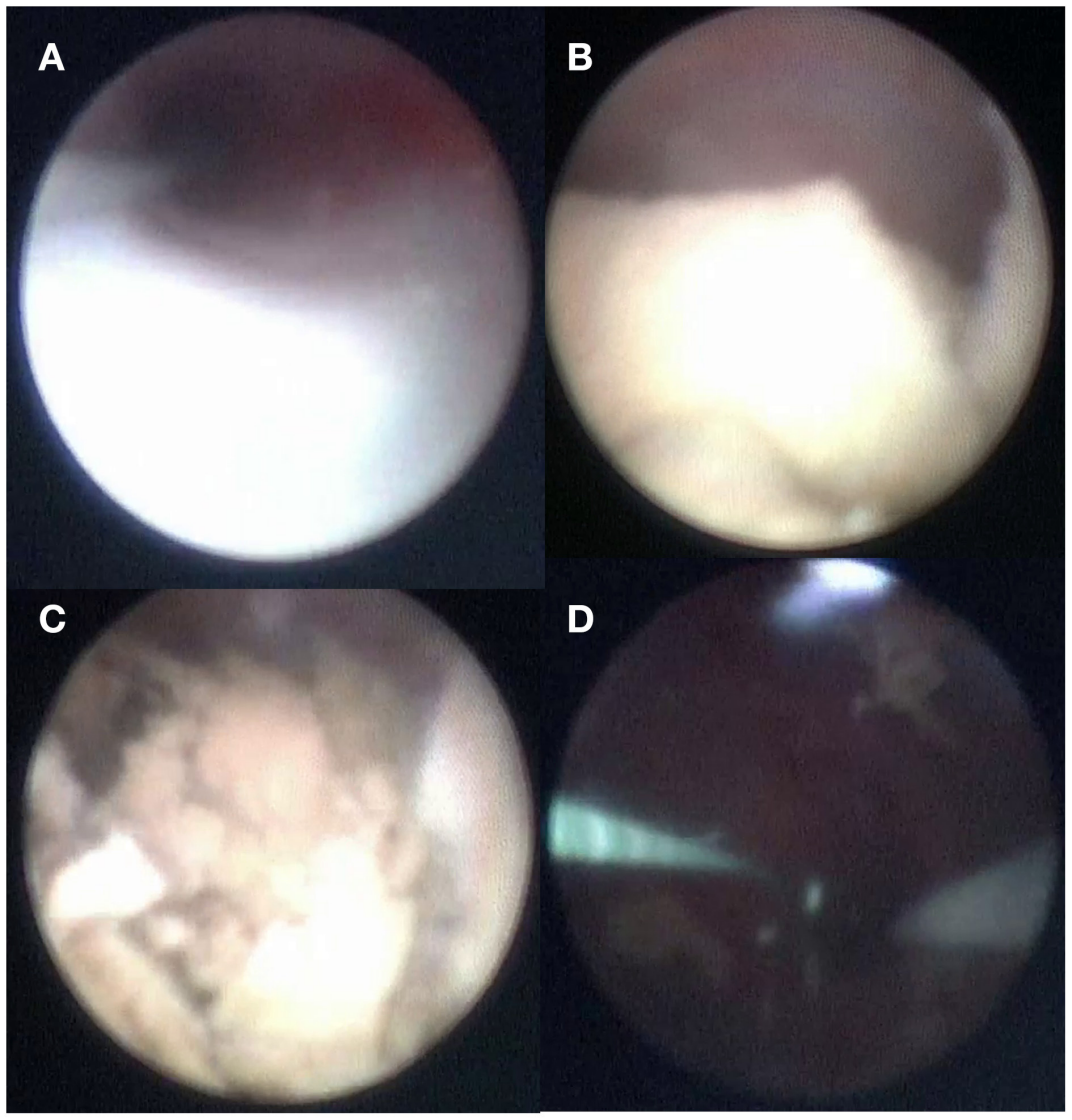
Microureteroscopy in patient 2. **(A)** Ureteroscopy. **(B)** Renal pelvis lithiasis. **(C)** Lithotripsy with Ho: YAG Laser. **(D)** Renal pelvis without lithiasis after treatment.

The lithotripter system used was Ho:YAG Laser with 200 μm fiber in all cases (Figure 3). Lithiasis were successfully pulverized during the procedure. Patient with coraliform stone, medium calyx lithiasis could be fragmented after producing a popcorn effect. However, lower calyx lithiasis could not be treated during this procedure.

## Results

Mean operating time was 100 ± 16 min. There were no intraoperative complications. One patient presented the intraoperative diagnosis of PUJO thanks to retrograde pyelography. In this case, a dilation of the pyeloureteral junction with a high pressure balloon was performed during the same procedure.

In all procedures, complete pulverization of the lithiasis was achieved, except in a girl with additional lithiasis in the middle and inferior calyx. In this case, a 5 mm residual stone remained in the lower calyx and an extracorporeal lithotripsy session was performed, reducing the residual lithiasis to 2 mm, which currently remains asymptomatic.

Se colocó doble J en el primer procedimiento masculino y la paciente femenina asoció PUJO, por dilatación con balón de alta presión de PUJ. In the first case, it was removed 2 months later, and the second case 4 weeks later.

All patients were discharged 24 h after the procedure. One of them was readmitted to the hospital 7 days after the intervention due to acute urine retention. The exploration revealed a lithiasic residue embedded in the urethral meatus that was removed with manual extraction. She was discharged after 24 h without complications.

Lithiasis composition was calcium phosphate in 2 patients and struvite in other. After more than 12 months of follow-up, all patients remain asymptomatic. Radiological and ultrasound scans described 2 mm residual lithiasis in the patient with additional lithiasis (middle and inferior calyx), being asymptomatic and without significant hydronephrosis in the other 2 children (Table 1).

**Table 1:** Patients data.

	**Patient 1 (boy)**	**Patient 2 (girl)**	**Patient 3 (girl)**
Age	12 months	2 years	3 years
Size	9 mm and 9 mm	23 mm	15 mm (Coraliform)
Side	Bilateral	Right	Left
Hydronephrosis	II and III	IV	II
UTI	Yes	Yes	Yes
Operative time	90 and 85 min	105 min	120 min
Other procedures	No	High-pressure balloon dilatation PUJO	Extracorporeal lithotripsy
Complications	No	Urine acute retention	No
Composition	Calcium phosphate	Struvite	Calcium phosphate
Residual lithiasis	No	No	2 mm

## Discussion

The management of kidney stones in children remains a challenge. The prevalence of pediatric lithiasis is increasing in all countries and we must perform treatments that reduce potential kidney damage. For this reason, minimally invasive treatments have become the gold-standard of treatment. Thus, the combination of some techniques similar to adults is used in our children patients: SWL, RIRS, PCNL, and open surgery if necessary (7). This allows a short hospital stay, good postoperative pain control, and low morbidity.

Recently, the appearance of smaller instruments has allowed new alternatives for the minimally invasive treatment of lithiasis. The “MicroPerc” system has been described as a new minimally invasive percutaneous nephrolithotomy technique that is performed with a 4.85 or 8 F metal needle. The technique was devised as a puncture system that allows access to the chosen calyx with vision at all times due to a fine optical fiber found in the needle (8). Desai and Mishra (3) proposed using the needle as a percutaneous access sheath, which was able to reduce puncture and subsequent sheath placement to a single step after dilating the percutaneous tract. To do this, they preferably used the 8 Fr metal needle. The technique was called “microperc” because it achieved percutaneous access below 10 Fr. Furthermore, the three-way connection attached to the needle allows a 272 μm laser fiber to be passed through the needle to perform lithotripsy or endopyelotomy. Lithotripsy was performed with the basic modalities of fragmentation and pulverization. Irrigation with saline solution and a 0.9 mm flexible fiber optic connected to the telescope are added to the other 2 connections for an adequate view, transmitting the image from the tip of the sheath. Some authors used the “microperc” access in a limited number of children for the treatment of lithiasis or recurrent peiloureteral stenosis, demonstrating that this technique can also be considered safe in preschool children (3, 5–9). However, there is no evidence of its efficacy in larger series of patients.

In some centers, instruments used in adults are also used for the management of lithiasis in children, which increases the potential ureteral damage when passing the ureteroscope. Although we use pediatric ureteroscopes, in preschool children it is difficult to perform ureteroscopy without previously dilating the meatus with a high-pressure dilatation balloon or weeks after placement of a double J stent that performs passive ureteral dilatation (10). The miniaturization of the equipment for percutaneous renal surgery allowed its use in distal ureteral stones in women with shorter urethra than in men. This reduces pain, ureteral damage, and the use of catheters after the operation. Thus, the recovery is faster.

In relation to this, it was decided to use of 4.8 F sheath of the MicroPerc device to diagnose and treat ureteral pathologies in small infants and children. The length of the sheath is 22 cm. Furthermore, the small length of the infant's urethra allowed us to easily achieve retrograde access to the renal pelvis.

Retrograde endoscopic procedures may present the risk of ureteral injury and difficult access in young children, requiring preliminary double-J stenting to increase the compliance of the urinary tract. We usually perform ureteral meatus dilatation during conventional ureteroscopy in young children. This retrograde renal access showed several advantages than the usual one with an ureteroscope: we did not need dilatation of the ureteral meatus nor the use of a temporary pre-stenting with double J to achieve distension of ureter. In this way, we reduce morbidity associated with renal access (11).

We placed double J in two procedures. In one of them, it was placed after pyelo-ureteral junction dilatation by high-pressure balloon due to associated PUJO. In the other case, the patient with bilateral lithiasis, the double J was placed during the first microureteroscopy due to our inexperience with this technique. He was our first patient treated with this approach. Furthermore, a new procedure under general anesthesia was not necessary that double J could be removed 2 months later during the contralateral microureteroscopy. However, we learned with this child that double J was not routinely necessary. We need larger series of patients to obtain more definitive conclusions on this matter.

The impossibility to access some middle calyzes and the lower calyx was our main technical difficulty. Likewise, the length of the 4.8 F sheat is small (22 cm), so access to the upper calyx will be difficult for older children. Therefore, adequate patient selection is crucial for a good outcome. We cannot forget that we are using the MicroPerc as a semi-rigid ureteroscope.

Unlike pediatric ureteroscopes, the microperc sheat is not telescoped. This makes it possible not to obstruct the ureteral meatus when ascending to the renal pelvis, thus reducing the damage caused by renal hyperpressure. Although the image quality is worse than modern videoureteroscopes it is acceptable during lithotripsy. Irrigation serum was passed around the sheath, avoiding high pressure.

Many authors use a safety guide during ureteroscopy. However, a safety guide was not usually used during our microureteroscopies. We did not used to place it to facilitate the access to the renal pelvis with the MicroPerc sheath. It was only used in one patient due to he presented PUJO that required high-pressure balloon dilatation. In this patient, the procedure was performed with hydrophilic guidance to avoid traumatizing the pyeloureteral junction. Obviously, we can use a safety guide if we consider it necessary in some patients without interfering with the procedure.

Unlike pediatric ureteroscopes, the micro-ureteroscopy sheath allows us to work with laser fibers between 230 and 270 microns. There are no conclusive studies that relate the size of the fiber with the time required to spray a stone (12). We can find difficulties in breaking some types of stones such as cystine. However, pediatric stones are usually softer than adult ones. In our case, we have not found any problem to eliminate stones in an acceptable period of time.

The MicroPerc equipment has a higher initial cost than a conventional ureteroscopy. Similar to other minimally invasive treatments, the initial cost must be offset by reducing the cost of operating room use, hospitalization, and complications. Furthermore, in our case, the minimum requirement of auxiliary procedures (transient nephrostomy or JJ ureteral stenting) reduced the total cost of the procedure. We think that we will also have less associated morbidity (2, 6).

In conclusion, we believe that microureteroscopy can be considered a new alternative for the treatment of selected cases of renal pelvic lithiasis in infants and small children. The feasibility of the technique has been demonstrated and no major complications have been found.

## Data Availability Statement

The original contributions presented in the study are included in the article/supplementary material, further inquiries can be directed to the corresponding author/s.

## Ethics Statement

The studies involving human participants were reviewed and approved by Instituto Investigación Sanitaria Gregorio Marañón. Written informed consent to participate in this study was provided by the participants' legal guardian/next of kin. Written informed consent was obtained from the minor(s)' legal guardian/next of kin for the publication of any potentially identifiable images or data included in this article.

## Author Contributions

AP is the surgeon responsible and the author of the text. RO has reviewed and contrasted the clinical data of the patients. BF-B and LB have revised the text and language. JA has directed the surgical procedures and the article. All authors contributed to the article and approved the submitted version.

## Conflict of Interest

The authors declare that the research was conducted in the absence of any commercial or financial relationships that could be construed as a potential conflict of interest.
